# Proteomics of Coagulopathy Following Injury Reveals Limitations of Using Laboratory Assessment to Define Trauma-Induced Coagulopathy to Predict Massive Transfusion

**DOI:** 10.1097/AS9.0000000000000167

**Published:** 2022-05-25

**Authors:** Hunter B. Moore, Matthew D. Neal, Marnie Bertolet, Brian A. Joughin, Michael B. Yaffe, Christopher D. Barrett, Molly A. Bird, Russell P. Tracy, Ernest E Moore, Jason L. Sperry, Brian S. Zuckerbraun, Myung S. Park, Mitchell J. Cohen, Stephen R. Wisniewski, James H. Morrissey

**Affiliations:** From the * Department of Surgery, University of Colorado, Denver, CO; † Department of Surgery, Pittsburgh Trauma Research Center, University of Pittsburgh, Pittsburgh, PA; ‡ Department of Epidemiology, University of Pittsburgh, Pittsburgh, PA; § Department of Biological Engineering, Massachusetts Institute of Technology, Cambridge, MA; ∥ Koch Institute for Integrative Cancer Research at MIT, Cambridge, MA; ¶ Center for Precision Cancer Medicine; # Department of Surgery, Beth Israel Deaconess Medical Center, Harvard Medical School, Cambridge, MA; ** University of Vermont, Department of Biochemistry, Burlington, VT; †† Department of Surgery, Ernest E Moore Shock Trauma Center at Denver Health, Denver, CO; ‡‡ Department of Surgery, Mayo Clinic Rochester, Rochester, MN; §§ Departments of Biological Chemistry and Internal Medicine, University of Michigan Medical School, Ann Arbor, MI.

**Keywords:** coagulopathy, massive transfusion, proteomics, trauma, trauma induced coagulopathy.

## Abstract

Trauma-induced coagulopathy (TIC) is provoked by multiple mechanisms and is perceived to be one driver of massive transfusions (MT). Single laboratory values using prothrombin time (INR) or thrombelastography (TEG) are used to clinically define this complex process. We used a proteomics approach to test whether current definitions of TIC (INR, TEG, or clinical judgment) are sufficient to capture the majority of protein changes associated with MT.

**Methods:**

Eight level I trauma centers contributed blood samples from patients available early after injury. TIC was defined as INR >1.5 (INR-TIC), TEG maximum amplitude <50 mm (TEG-TIC), or clinical judgment (Clin-TIC) by the trauma surgeon. MT was defined as >10 units of red blood cells in 24 hours or >4 units RBC/hour during the first 4 hours. SomaLogic proteomic analysis of 1305 proteins was performed. Pathways associated with proteins dysregulated in patients with each TIC definition and MT were identified.

**Results:**

Patients (n = 211) had a mean injury severity score of 24, with a MT and mortality rate of 22% and 12%, respectively. We identified 578 SOMAscan analytes dysregulated among MT patients, of which INR-TIC, TEG-TIC, and Clin-TIC patients showed dysregulation only in 25%, 3%, and 4% of these, respectively. TIC definitions jointly failed to show changes in 73% of the protein levels associated with MT, and failed to identify 26% of patients that received a massive transfusion. INR-TIC and TEG-TIC patients showed dysregulation of proteins significantly associated with complement activity. Proteins dysregulated in Clin-TIC or massive transfusion patients were not significantly associated with any pathway.

**Conclusion:**

These data indicate there are unexplored opportunities to identify patients at risk for massive bleeding. Only a small subset of proteins that are dysregulated in patients receiving MT are statistically significantly dysregulated among patients whose TIC is defined based solely on laboratory measurements or clinical assessment.

## INTRODUCTION

Trauma-induced coagulopathy (TIC) is associated with high-blood product utilization and increased mortality.^1^ However, clinical and laboratory definitions of this process, and associated molecular drivers, are debated.^2,3^ Innes and Sevitt in 1964 were among the first to document that prothrombin time is prolonged following severe injury.^4^ Forty years later, prolongation of prothrombin time after injury was identified as an independent predictor of mortality in two separate trauma systems.^5,6^ These observations gave rise to defining coagulopathy in trauma using the international normalized ratio of prothrombin time (INR).^7–9^ Around this time, the cell-based model of coagulation stimulated interest in assessing coagulation beyond the plasma-based INR.^10^ Interest in whole-blood coagulation assessment in trauma patients using viscoelastic hemostatic assays evolved rapidly.^11^ These assays, including thrombelastography (TEG) and rotational thrombelastometry (ROTEM), are now used to guide resuscitation and blood product transfusion in large volume trauma centers. Like INR, a hypocoaguable viscoelastic measurement of maximum amplitude (MA, clot strength) has been associated with increased mortality in trauma.^12^ This new definition of coagulopathy following trauma was corroborated at trauma centers throughout the world.^13,14^

Correlation between abnormal viscoelastic parameters and INR measurements to define coagulopathy is lacking.^15^ In addition, depletion of coagulation factors is insufficient to explain why trauma patients have prolongation of the prothrombin time.^16^ Neither of these laboratory-based definitions of TIC have taken into consideration the patient’s clinical level of coagulopathy, which has only recently been defined with a stratified score.^17^ With growing evidence that TIC is a multifactorial process^1^ with temporal variability,^2^ the Trans-Agency Consortium for Trauma-Induced Coagulopathy (TACTIC) investigated if a single laboratory value or clinical judgment was sufficient to define trauma-induced coagulopathy related to massive bleeding. We performed a proteomic analysis of trauma patient samples obtained in the emergency department from participants at risk for hemorrhage and TIC who were enrolled in TACTIC associated clinical trials. Considering the diverse phenotypes of TIC, we hypothesized that the different definitions of trauma-induced coagulopathy (INR, TEG, or clinical judgment) will have discordant proteome pathway analyses and will not capture the majority of protein changes associated with massive bleeding in trauma.

## METHODS

### Patient Population and TIC Definitions

Candidates for this study were a subgroup of participants in the three TACTIC associated studies (Control of Major Bleeding after Trauma—COMBAT, Prehospital Air Medical Plasma Trial—PAMPer, and Study of Tranexamic Acid during Air Medical Prehospital Transport—STAAMP), and a prospective observational coagulation assessment study from the Mayo Clinic, using methods published previously.^18–20^ Trauma patients (n = 211) from eight level I trauma centers were identified for this analysis. Patients were eligible if they were >18 years of age, had available stored blood samples from emergency department arrival, received more than one unit of red blood cells (RBC), and survived >24 hours. Traumatic brain injury was defined as CT imaging confirming structural brain injury.

Three definitions of TIC were developed and compared. INR-TIC was defined as INR > 1.5 at emergency department admission, based on the original threshold proposed by Brohi et al^5^ and a subsequent multicenter study demonstrating that an INR > 1.5 was an independent predictor for mortality, whereas lower thresholds lost significance after risk adjustment.^21^ The second definition was TEG-MA < 50 mm (TEG-TIC) at admission. Massive transfusion and increased mortality have previously been associated with clot strength in this range.^13,14,22–24^ Clinical coagulopathy (Clin-TIC) was defined by the prospective evaluation of the treating physician’s determination in the emergency department that the patient had coagulopathic bleeding requiring preemptive hemostatic blood product resuscitation before laboratory results were available (corresponding to a bleeding score of greater than 3 using the TACTIC scoring system).^17^

### Samples and SOMAscan Analysis

Blood collection occurred at emergency department admission. Collection tubes (SCAT-144; Haematologic Technologies, Essex Junction, VT) contained EDTA as anticoagulant, and multiple protease inhibitors to eliminate *ex vivo* proteolytic activity (4-(2-Aminoethyl) benzenesulfonyl fluoride hydrochloride (AEBSF), 0.5 mM; Aprotinin, 300 KIU/mL; Elastatinal, 20 μM; GGACK, 10 μM; E-64, 5.0 μM; Pepstatin A, 1.0 μM).

Samples were sent to SomaLogic for analysis using the SOMAscan v. 1.3k platform. Briefly, the SOMAscan assay uses aptamers (~40-mer oligonucleotide strands with proprietary side chains) selected for binding to human plasma proteins and protein complexes, allowing quantification of >1300 proteins levels in parallel^25^. The v. 1.3k assay measures a broad range of secreted proteins, markers of cell death, hormones, growth factors, cytokines, and so on. The assay signal is expressed as relative fluorescence units (RFU). In addition to the RFU data, SomaLogic also performed a normalization using internal standard samples, and provided normalized data. After quality control, data were provided on 1305 proteins. The relation of SOMAscan data to data collected with more traditional assays has recently been explored.^26^ Although some differences are emerging, many proteins yield consistent results across multiple platforms.

### Outcomes

Hemorrhage is the most common cause of preventable death in trauma^27,28^ with massive transfusion (MT)^29,30^ being a dichotomous outcome associated with numerous definitions of TIC.^1,2^ Two definitions of MT based on red blood cell (RBC) transfusions were combined to capture the maximum number of patients actively bleeding following injury. The first definition included a temporal component of greater than 4 units of blood products over the course of one hour (MT/Hr) within the first 4 hours of arriving at the emergency department.^29^ The second definition was based on total volume over a 24-hour period [>10 units RBC (MT/24hr)].^30^ Both of these definitions of massive transfusion have clinical relevance, as they would require multiple coolers of blood products (typically 4 units of RBC paired with thawed plasma) to resuscitate a trauma patient. Whereas patients below these thresholds could be resuscitated without thawing multiple blood products pre-emptively and could be resuscitated with blood products immediately available upon patient arrival to the hospital.

### Statistical Methods

SomaLogic provided normalized and non-normalized datasets for plasma samples collected at time of presentation at the emergency department. The normalized data were utilized per company recommendations. Clinical and laboratory data from each of the three TACTIC studies were sent to the TACTIC Data Coordinating Center at the University of Pittsburgh and harmonized according to criteria agreed upon by the trial’s principal investigators. Participants had their citrated rapid TEG-MA values (n = 173 of 211) and clinical INR lab (n = 203 of 211) from emergency department arrival as their baseline values. The clinical TIC score was collected by each study and was available for 181 of 211 participants. For each of the 1305 SOMAscan analytes, a Kruskal-Wallis nonparametric test was performed to determine if there was a difference between those with and without TIC according to each criterion using INR-TIC, TEG-TIC, Clin-TIC score, and massive transfusion status. For each of these criteria, the false discovery rate (FDR) using the linear set-up method was determined^31^ and those proteins with a resulting FDR adjusted *P* value <0.05 were classified as significantly different for that criterion. This analysis is provided as Table S1 (http://links.lww.com/AOSO/A123) (and for unnormalized SOMAscan data as Table S2, http://links.lww.com/AOSO/A124, although this was not used in this study). The proteins that differed significantly in patients with the TIC criteria were compared with the proteins that significantly differed in patients according to the massive transfusion status. This analysis was performed in SAS 9.4 (SAS Institute Inc., Cary, NC).

### Pathway Analysis

A platform file describing the SOMAscan Assay 1.3k analytes was downloaded from Gene Expression Omnibus (https://www.ncbi.nlm.nih.gov/geo/query/acc.cgi?acc=GPL23119). From this, a table of aptamers and their associated analytes was extracted. For each aptamer’s target analyte proteins, a single most representative gene name was chosen by manual annotation to prevent complexes detected by SOMAscan, multiple analytes with similar functional annotations detected by a single aptamer, from dominating downstream analysis. This manual annotation is provided as Table S3 (http://links.lww.com/AOSO/A125). From this list of one gene per SOMAscan aptamer, five lists of unique gene names were made: all genes encoding SOMAscan target proteins, genes encoding SOMAscan target proteins significantly dysregulated in patients who received MT, and genes encoding SOMAscan target proteins significantly dysregulated in patients positive for each of the three definitions of TIC (INR-TIC, TEG-TIC, or Clin-TIC).

Dysregulation in each case was indicated by significant difference in distribution in the timepoint-normalized data set, without regard to the sign of the difference. These lists are provided in Table S4 (http://links.lww.com/AOSO/A126) and were uploaded as UniProt^32^ annotations to version 6.8 of the database for annotation, visualization and integrated discovery (DAVID).^33^ Functional annotations of the list of SOMAscan target proteins were compared with those of the whole human genome, and annotations of the lists of dysregulated proteins was compared with that of SOMAscan target proteins, using DAVID with default annotation categories. Individual functional annotations were ranked by Benjamini-Hochberg-corrected FDR^34^ and clusters of annotations were ordered by the enrichment score provided by DAVID. Functional annotation term names and categories were manually edited for clarity in Table 3 and Table S6 (http://links.lww.com/AOSO/A128) but are unedited in Table S5 (http://links.lww.com/AOSO/A127). Terms enriched in each set were also clustered by DAVID on the basis of terms that annotate similar sets of proteins in common. These cluster descriptions are also included in Table S3 (http://links.lww.com/AOSO/A125).

## RESULTS

### Patient Population

Patients were predominantly male (70%) with an average age of 45 years. The majority of patients had blunt injury (82%), were severely injured (average ISS 24), and sustained multisystem trauma (79%) with roughly a third having a traumatic brain injury (30%, Table 1). The overall cohort had median shock index of 1.4 in the emergency department supporting a hemodynamically unstable patient population. The majority of these patients had an INR (96%) and TEG (82%) drawn in the emergency department. There was evidence of coagulation abnormalities in the majority of the patient cohort with the group having an average INR of 1.5, average TEG-MA of 56.3, and 25% were clinically deemed to be clinically coagulopathic. The patient population had a 22% rate of massive transfusion and mortality rate prior to discharge of 12%. Within the massive transfusion cohort, the median number of RBCs transfused was 11 units compared with the non-MT group at 3U (*P* < 0.0001)

**TABLE 1.** Patient Demographics, Injury Patterns, and Outcomes

Characteristics	Total (N = 211)	INR > 1.5 (N = 30) INR-TIC	INR <1.5 (N = 173)	*P*	MA < 50 (N = 37) TEG-TIC	MA > 50 (N = 136)	*P*	Clin Yes (N = 46) Clin-TIC	Clin No (N = 135)	*P*
Patient demographics										
Male, n (%)	147 (70%)	23 (77%)	119 (69%)	0.385	28 (76%)	96 (71%)	0.524	35 (76%)	92 (68%)	0.310
Age, mean, SD	45, 18	36, 18	47, 17	0.001	42, 20	46, 17	0.174	47, 21	45, 17	0.587
Injury pattern and severity										
ISS, mean, SD	24, 13	28, 15	24, 12	0.062	27, 12.3	25, 12.4	0.351	28, 12	25, 13	0.092
Blunt Injury, n (%)	172 (82%)	21 (70%)	145 (83%)	0.070	28 (76%)	112 (82%)	0.359	38 (83%)	110 (82%)	0.864
TBI, n(%)	63 (30%)	8 (27%)	55 (32%)	0.575	11 (30%)	44 (32%)	0.761	13 (28%)	45 (33%)	0.524
Polytrauma, n (%)	166 (79%)	22 (73%)	138 (80%)	0.390	30 (81%)	113 (84%)	0.706	38 (83%)	113 (84%)	0.784
Shock index, median Q1,Q3	1.4 (1.1–1.7)	1.6 (1.3–2.0)	1.3 (1.1–1.7)	0.047	1.5 (1.0–1.9)	1.3 (1.1–1.7)	0.236	1.6 (1.3–1.9)	1.3 (1.2–1.7)	0.012
Definitions of TIC										
INR-TIC, n (%)	30 (15%)	30 (100%)	0 (0%)	Na	14 (38%)	16 (12%)	<0.001	12 (27%)	14 (11%)	0.010
TEG-TIC, n (%)	37 (21%)	14 (47%)	23 (16%)	<0.001	37 (100%)	0 (0%)	Na	19 (42%)	17 (14%)	<0.001
Clin-TIC, n (%)	46 (25%)	12 (46%)	33 (22%)	0.010	19 (53%)	25 (19%)	<0.001	46 (100%)	0 (0%)	na
Outcomes										
MT hour, n (%)	41 (19%)	8 (27%)	31 (18%)	0.262	11 (30%)	28 (21%)	0.238	22 (47%)	19 (14%)	<0.001
MT 24 h, n (%)	26 (12%)	6 (20%)	19 (11%)	0.165	9 (24%)	15 (11%)	0.038	16 (35%)	9 (7%)	<0.001
Composite MT, n (%)	47 (22%)	11 (37%)	34 (20%)	0.038	14 (38%)	31 (23%)	0.064	25 (54%)	21 (16%)	<0.001
Total Number RBC, mean, SD	6.1,5.5	6.7,5.6	5.9,5.5	0.5415	8.3,7.6	5.5,4.7	0.088	11.6,5.9	3.3,2.1	0.004
Mortality, n (%)	24 (12%)	6 (20%)	18 (11%)	0.143	7 (19%)	14 (10%)	0.144	7 (16%)	15 (11%)	0.451

Clin-TIC, clinical assessment of coagulopathy; composite MT, MT and or MT 24 hours; INR-TIC, International normalized ratio of prothrombin time definition of coagulopathy; ISS, injury severity score; MT 24 hrs, >10 units red blood cells per hour in 24 hours; MT hour, >4 units of red blood cell units per hour within the first 6 hours; TBI, traumatic brain injury; TEG-TIC, TEG definition of coagulopathy.

### Clinical Variables of TIC Cohorts and Patients Undergoing Massive Transfusion

**FIGURE 1.**
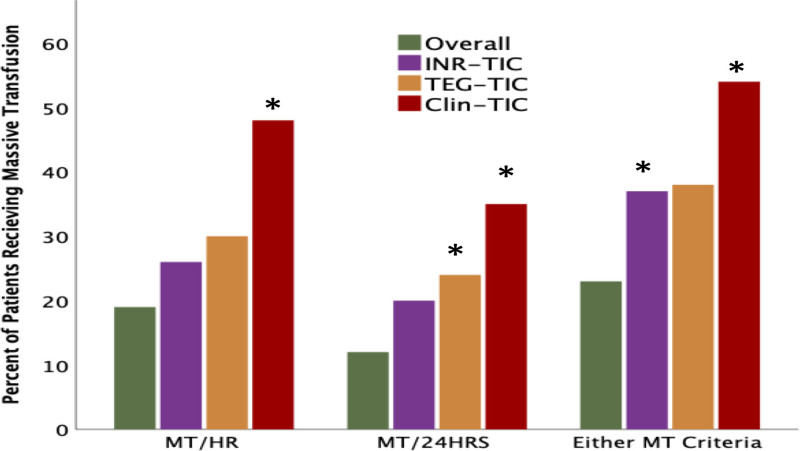
Rates of massive transfusion stratified by TIC definition. MT/Hr indicates >4 units of red blood cell units per hour; MT/24hrs, >10 units red blood cells per hour in 24 hours; either MT criteria; MT/Hr and or MT/24hrs. INR-TIC, international normalized ratio of prothrombin time definition of coagulopathy; Clin-TIC, clinical assessment of coagulopathy; TEG-TIC, TEG definition of coagulopathy.

TIC definitions were not specific to any patient demographics with the exception of younger age associated with INR-TIC (Table 1). Injury pattern and severity also did not significantly associate with any TIC definition. However, an increased shock index was associated with an INR-TIC, Clin-TIC. Clin-TIC was significantly associated with all the two definitions and MT, whereas TEG-TIC was only associated with 24 hours MT, and INR-TIC was only significant for the composite MT score (Figure 1). If a patient met one or more TIC definitions the rate of MT was 74%. Patients with TEG-TIC versus non-TEG-TIC and INR-TIC versus non INR-TIC had almost twice as many deaths after 24 hours compared with non-TEG-TIC and non INR-TIC patients, whereas Clin-TIC versus non-Clin-TIC had comparable mortality rates (Table 1).

### SOMAscan Analysis of Massive Transfusion and TIC Definitions

**FIGURE 2.**
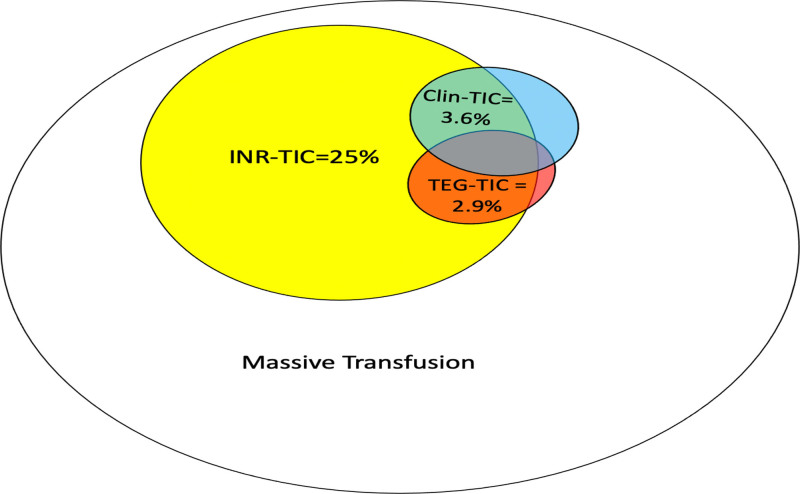
Proteome inclusion of TIC definition of proteins associated with massive transfusion. It is a graphic representation of the proteins that were significantly associated with massive transfusion and the component captured by the different definitions of TIC. Each TIC definitions protein percentages and overlap are represented by different colored ovals. Overall, only 27% of proteins associated with massive transfusion were captured by the three different definitions of TIC.

SOMAscan analysis of 1305 proteins identified significant differences in 578 analytes between MT and non-MT patients. Proteins associated with MT and TIC definitions (normalized data set) are provided in Table S1 (http://links.lww.com/AOSO/A123). INR-TIC status was associated with a significant difference in 147 (25%) of these, with 73 additional analytes associated with an elevated INR and not massive transfusion. Patients with TEG-TIC significantly differed from patients with non-TEG-TIC patients on 17 (2.9%) analytes dysregulated in MT patients, along with an additional 7 protein analytes that did not differ with massive transfusion status. Patients with the Clin-TIC definition showed dysregulation in 21 proteins (3.6%) that also differed with massive transfusion, as well as one protein that did not. The majority of analytes dysregulated in patients who received massive transfusions (73% Figure 2) were not captured by any definitions of TIC.

**FIGURE 3.**
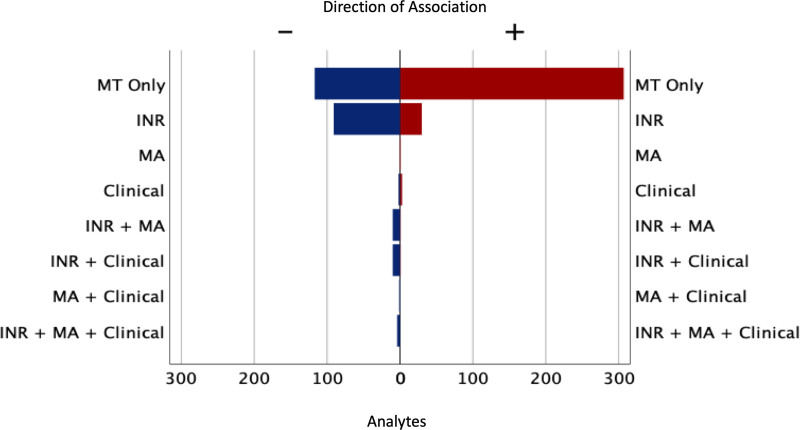
Directional association of analytes associated with massive transfusion and TIC. MT, >4 units of red blood cell units per hour and or 10 units red blood cells per hour in 24 hours; INR-TIC, International normalized ratio of prothrombin time definition of coagulopathy; Clin-TIC, Clinical assessment of coagulopathy; TEG-TIC, TEG definition of coagulopathy.

Figure 3 demonstrates the distribution of analytes dysregulated in association with massive transfusion and with the different TIC definition combinations. There were 4 analytes that significantly varied in patients based on all three TIC definitions and with MT, of which all were downregulated (Table 2). TEG-TIC and INR-TIC shared 11 analytes associated with MT. All of the analytes associated with MT and TEG+INR-TIC were decreased, with the exception of hemoglobin (Table 2). Clin-TIC and INR-TIC shared 11 analytes associated with MT, all of which were down regulated, with exception of pulmonary surfactant-associated protein D (Table 2). Decreased levels of vascular endothelial growth factor receptor 2 was the only protein associated with TEG-TIC, Clin-TIC, and MT.

**TABLE 2.** TIC Definitions and Associated Proteins

Definition	Protein	Direction of Association
INR+TEG+Clin+MT	Apolipoprotein E	–
INR+TEG+Clin+MT	Bone morphogenetic protein 1	–
INR+TEG+Clin+MT	Growth hormone receptor	–
INR+TEG+Clin+MT	Complement C5b-C6 complex	–
INR+TEG+MT	Hemoglobin	+
INR+TEG+MT	Protein FAM107B	–
INR+TEG+MT	Complement C3b	–
INR+TEG+MT	Complement component C5	–
INR+TEG+MT	Tumor necrosis factor receptor superfamily member 3	–
INR+TEG+MT	Complement component C8	–
INR+TEG+MT	Alpha-(1,3)-fucosyltransferase 5	–
INR+TEG+MT	Vitamin K-dependent protein S	–
INR+TEG+MT	Muellerian-inhibiting factor	–
INR+TEG+MT	Neurexophilin-1	–
INR+TEG+MT	Haptoglobin	–
INR+Clin+MT	Pulmonary surfactant-associated protein D	+
INR+Clin+MT	Parathyroid hormone-related protein	–
INR+Clin+MT	Interleukin-37	–
INR+Clin+MT	Tyrosine-protein kinase receptor TYRO3	–
INR+Clin+MT	Phosphoglycerate mutase 1	–
INR+Clin+MT	Proprotein convertase subtilisin/kexin type 7	–
INR+Clin+MT	Nicotinamide phosphoribosyltransferase	–
INR+Clin+MT	Interleukin-19	–
INR+Clin+MT	Plasma kallikrein	–
INR+Clin+MT	C5a anaphylatoxin	–
INR+Clin+MT	Complement C1r subcomponent	–
TEG+Clin+MT	Vascular endothelial growth factor receptor 2	–

Clin, Clinical assessment of coagulopathy; INR, International normalized ratio of prothrombin time definition of coagulopathy; MT, > 4 units of red blood cell units per hour and or 10 units red blood cells per hour in 24 hours; TEG, TEG definition of coagulopathy.

There were 121 analytes that were uniquely associated with dysregulation in INR-TIC and MT patients (Table S1, http://links.lww.com/AOSO/A123). The majority of these proteins (74%) were associated with decreased levels in both INR-TIC and MT (Figure 3). There were 8 analytes associated with dysregulation in Clin-TIC and MT (Table 2). There were 424 analytes captured associated with dysregulation in MT patients that were not associated with any dysregulation in patients in the populations defined by TIC, of which 307 (72%) were increased in MT (Figure 3 and Table S1 [http://links.lww.com/AOSO/A123]). The MT-dysregulated proteins not captured by TIC definitions missed several key regulators of fibrinolysis including increased levels of tissue plasminogen activator and decreased levels of alpha 2-antiplasmin, and carboxypeptidase B2 [thrombin activatable fibrinolysis inhibitor (TAFI)] (Table S1, http://links.lww.com/AOSO/A123).

### Pathway Analysis

To discover whether the SOMAscan target plasma proteins dysregulated in patients who had various indications of TIC differed from each other and from those dysregulated in patients who received a massive transfusion, the functional annotations of these proteins were compared with those of all SOMAscan targets using the bioinformatics tool DAVID^33^ (see Methods). SOMAscan targets themselves have significant differences in their annotations than the background of the whole proteome. Clusters of terms pertaining to secretion, glycosylation, immunity, kinases, and proteases, and many others are significantly enriched in SOMAscan analytes relative to the proteome (Table S5, http://links.lww.com/AOSO/A127). For this reason, it was important that proteins associated with TIC were compared not to the whole proteome, but to the subset that SOMAscan measures.

**FIGURE 4.**
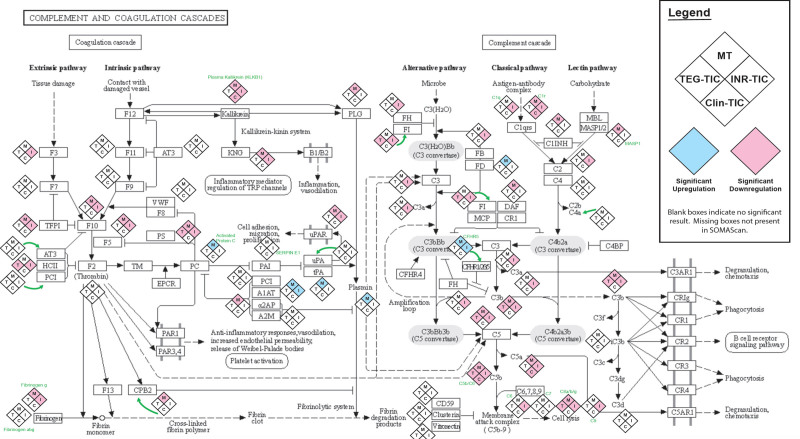
Schematic of pathway regulation of coagulation and complement related to different definitions of TIC and massive transfusion.

Relative to the background of all SOMAscan analytes, the complement pathway was the only process/pathway enriched, and was only associated with the sets of proteins dysregulated in INR-TIC and TEG-TIC patients (Table 3). Proteins dysregulated among patients who received MTs are significantly enriched only for general functional annotations (not pathways or processes) related to membrane localization or glycosylation. Functional annotations not associated with a particular pathway, but significantly enriched within each group (FDR ≤ 0.1), are listed in Table S6 (http://links.lww.com/AOSO/A128). No terms were significantly enriched among proteins dysregulated in patients diagnosed with TIC by clinical judgement. Figure 4 shows the KEGG pathway^35^ depicting the complement and coagulation cascades, annotated by which proteins are significantly up- and downregulated among patients who received MTs, or with different definitions of TIC.

**TABLE 3.** Enrichment of Functional Annotations of Pathways and Processes Dysregulated Among Patients With Different Indications of TIC

Functional Annotation	Category	List Hits^*^	List %^*^	Pop Hits^†^	Pop %^†^	Fold Enrichment	FDR^‡^
Massive transfusion							
None							
INR-TIC							
Complement and coagulation cascades	KEGG pathway	22	10.4%	47	5.5%	2.59	0.0020
Complement pathway	BioCarta	9	4.2%	15	3.6%	3.54	0.077
Classical complement pathway	BioCarta	8	3.8%	11	2.7%	4.29	0.079
TEG-TIC							
Regulation of complement activation	GO term: biological process	5	21.7%	18	1.4%	15.76	0.050
Clinical TIC							
None							

*List hits, list %: Number and fraction of proteins dysregulated among patients with the given indication of TIC that have the given annotation.

†Pop hits, pop %: Number and fraction of proteins that are targets of SOMAscan aptamers with the given clinical indication of TIC that have the given annotation.

‡FDR: All pathway/process terms with a false discovery rate of less than 0.1 are reported here. Nonpathway terms with FDR < 0.1 are reported in Table S6 (http://links.lww.com/AOSO/A128). Complete results are reported in Table S5 ((http://links.lww.com/AOSO/A127)).

## DISCUSSION

When evaluating a cohort of severely injured patients requiring blood product resuscitation, three different definitions of TIC had variable associations with massive transfusion (Figure 1). Proteomic analysis indicated the majority of proteins significantly different among patients requiring massive transfusion were not captured from the three definitions of coagulopathy (Figure 2). Analytes dysregulated in patient populations associated with TIC definitions and massive transfusion were predominantly decreased compared with patients who did not undergo a MT or meet a TIC definition (Figure 3). When conducting a pathway analysis, complement was associated with TIC definitions and MT (Figure 4), while the extrinsic and intrinsic coagulation cascade lacked a pattern associated with dysregulation. The analytes dysregulated in patients requiring massive transfusion and not captured by TIC definitions included regulators of the fibrinolytic system (Figure 4; Table S1, http://links.lww.com/AOSO/A123) and were associated with glycoproteins and membrane-associated proteins in the functional analysis (Table S6, http://links.lww.com/AOSO/A128).

Laboratory derangements in coagulation following trauma have been appreciated for decades,^4^ but clinical observation of bleeding abnormalities following trauma have been known for centuries.^36^ Both clinical assessment of TIC^17^ and laboratory detection of coagulopathy with TEG-TIC and INR-TIC identify patients who have an increased risk of MT, which has been reported previously.^5–9,11–14,21,23^ Attempting to harmonize a single laboratory measurement to define TIC remains elusive.^1,2^ In addition to the cluster of proteins common to all three TIC definitions (Table 2), each TIC definition had subtle differences in the complement and coagulation pathways that were up- and downregulated (Figure 4). Prior work in TIC has suggested that there are multiple phenotypes of TIC,^37–39^ which is consistent with this observation. Among the three definitions of coagulopathy INR-TIC captured a larger number of proteins associated with massive transfusion, while TEG-TIC and Clin-TIC overlapped to lesser degrees (Figure 2). However, a large number of analytes associated with major bleeding were not associated with the three TIC definitions. Fibrinolysis activation, for example, was not captured by the different TIC definitions but is associated with massive transfusion.

The drivers of clot formation and fibrinolysis have previously been reported to be divergent in TIC.^37,39^ These data support this concept as INR-TIC and TEG-TIC are related to clot generation, rather than clot degradation. TEG LY30 was not included as a TIC definition in this analysis despite its association with massive transfusion^40–42^ due to conflicting literature that viscoelastic testing lacks sensitivity to quantify fibrinolysis activity.^43,44^ Prior proteomics analysis of hyperfibrinolytic trauma patients, defined by TEG LY30, identified an expected pattern of depletion of plasminogen and alpha 2-antiplasmin.^45^ Another study using viscoelastic testing to define fibrinolysis dysregulation, confirmed the same depletion of TAFI, alpha 2-antiplasmin, and plasminogen with an increase in t-PA.^46^ The same pattern is appreciated in patients with massive transfusion, but not captured by the TIC definitions (Figure 4). These data suggest that fibrinolysis activity measurements should be incorporated in defining TIC, since INR, TEG-MA, and clinical judgment did not identify fibrinolysis that was associated with massive bleeding.

The drivers of impaired clot generation in TIC remain debated.^1,2^ Although employing a large number of multiple comparisons on data sets has been referred to as “fishing,”^47^ high-output biologic analysis is being used increasingly frequently and an alternative terminology has evolved as hypothesis evaluation (rather than the classical hypothesis testing) and has its role in research.^48^ These data provided an opportunity to perform a hypothesis evaluation of proteins associated with INR-TIC. Activated protein C (APC) was one of the first mechanisms proposed to drive impaired thrombin generation in trauma via presumed depletion of factors Va and VIIIa.^7^ Protein C was one of several SOMAscan analytes identified as dysregulated in patients requiring massive transfusion but not among patients identified by either of the TIC definitions including INR. APC has been implicated in an animal model of TIC,^49^ and several clinical studies measuring this protein have associated activation of protein C with an elevated INR and massive transfusion.^7,50^ In the pathway analysis coagulation factor V was depleted in INR-TIC and MT patients, in addition to protein S (cofactor for protein C), but depletion of factor VIII and activator of thrombomodulin was not appreciated (Figure 4), which has been proposed to be integral in driving INR-TIC.^7^ These data suggest that APC is associated with massive bleeding and not necessarily associated with a prolonged INR and supports the controversy that APC is not the central driver of TIC.^51^

Complement emerged as a potential driver of INR-TIC, as this was the only biological pathway associated with laboratory detected coagulopathy (Table 3). Complement has been implicated in driving impaired hemostasis.^52^ Conversely, the downregulation of many complement regulatory proteins observed here, despite consistent literature reporting elevated levels of complement anaphylatoxins and the terminal complement complex C5b-9 in trauma,^53–55^ support the growing hypothesis that coagulation and fibrinolysis proteases (eg, plasmin, factor Xa) may be the principal drivers of elevated complement end-products via pathway cross-talk.^56–58^ Factor X can also activate complement,^56^ and blood clotting with neutrophil priming appears to have a synergistic effect in complement activation.^54^ Depletion of coagulation factor X was associated with INR-TIC, which has been appreciated in other trauma studies, where coagulopathy was analyzed with proteomic analysis.^45,46^ Protein S, which has been reported to associate with factor VIII to decrease the rate of intrinsic factor X activation^59^ and also to modulate the activity of the anticoagulant, TFPI,^60^ so it is of note that protein S was depleted in the pathway analysis in INR-TIC and MT. It remains unclear from our data whether factor X and complement are causing prolongation in INR or are an associated biomarker of injury severity. Future work is needed to understand this relationship.

There are additional proteins associated with coagulopathic bleeding in trauma that were associated with massive transfusion in our analysis but lacking with INR-TIC and TEG-TIC. Damage associated molecular pattern proteins (DAMPs) are an example. DAMPs have been associated with hemorrhagic shock in proteomic analysis of an animal model.^61^ Specific to this study, high mobility group box –1 (HMGB-1) and histone-related proteins were elevated in patients undergoing massive transfusion in our proteomic analysis but were not associated with any of the TIC definitions, similar to protein C. HMGB-1 and histones have been shown to orchestrate platelet activation,^62,63^ and this has been associated with coagulopathy and poor outcomes following trauma.^64,65^ HMGBI and histones have been associated with an elevated INR in trauma,^64,65^ but the sensitivity of SOMAscan analytes may not be equivalent to gold standard ELISAs. Future work is needed to validate if these observed proteins associated with massive transfusion are not reliably captured with current clinical assays (INR and TEG) as DAMPs have been implicated from a mechanistic level to drive coagulation changes that could be missed in clinical practice.

One future clinical translation of these findings is to prospectively validate if additional biomarkers can be used to risk stratify patients for massive bleeding. Table S1 (http://links.lww.com/AOSO/A123) lists 424 proteins that are potential targets. With emerging technologies, the identification of specific antigens levels is feasible within minutes. Although identification of patients at risk of massive transfusion with a rapid point of case device will not treat the underlying cause, it at least would aid the optimal triage for treatment of massive hemorrhage, which is a resource intensive process. Further, although the evolution of rapid care technology based on these findings would be ideal, evolving evidence suggests that proteomic analyses can reliably cluster patients as responders or nonresponders to therapy.^65^ The findings in this work may be applied to future understanding of interventions in TIC to determine protein signatures of patients who are most likely to benefit from an intervention.

A limitation of all work in trauma-induced coagulopathy is a lack of consensus on a gold standard definition of TIC due to its complex nature.^2^ Massive transfusion is a proxy for uncontrolled bleeding, but dependent on obtaining both surgical control of and correction of coagulopathy. The nonmassive transfusion cohort did receive blood products, and there was a spectrum of how many units of blood these patients received. Dichotomizing patients to a specific outcome, with a continuous variable is not ideal, but the same limitation is applicable to using INR and TEG-MA to define TIC. In the non-MT group a median of 3 units of blood were transfused compared with 14 in the MT group, which demonstrates a large difference in blood product utilixation. Ultimately the goal of trauma resuscitation is to stop bleeding, and fixing a laboratory generated number remains debated if outcomes in trauma can be improved.^67,68^ Surgical control of bleeding as emphasized in the STOP THE BLEED initiative,^69^ and prompt resolution of shock are as essential as treating coagulopathy. Attenuation of coagulopathy may not have prevented the majority of these patients from receiving a massive transfusion with our current transfusion practices, and the question has been proposed as to whether these patients are bleeding because they are dying or dying from bleeding.^70^ Regardless of cause or association of these biomarkers, identifying patients at risk of bleeding in trauma remains a clinical challenge. Only half of the patients received a unit or more of blood in the CRASH II trials, which was one of the largest randomized control trials to treat bleeding in trauma.^71^ The same is true in more recent randomized control trials that used prehospital physiologic measurements to predict who was at risk of major bleeding, but only captured 1 in 4 patients that required a massive transfusion.^18,19^ Furthermore, the coagulopathy of traumatic brain injury (TIC) appears to be mechanistically distinct and, therefore, may require a unique definition of coagulopathy.

Additional limitations include the complexities in analyzing plasma proteomics, including the methods by which plasma is preserved and analyzed,^72^ and informatics challenges in analysis of thousands of proteins in hundreds of patients.^46^ We analyzed these data using both a descriptive and bioinformatic approach with DAVID,^33^ which clusters proteins based on previous work in coagulation and other cellular processes unrelated to trauma. Specific biological processes in response to trauma characterized by proteomics is evolving and definitive protein clustering is not possible. There were several protein key terms identified in DAVID [glycoproteins, glycosylation, microparticles (Table S6, http://links.lww.com/AOSO/A128)] associated with massive transfusion. These analyte groupings are consistent with proteins characterized as the “endotheliopathy” of trauma and could represent biological pathways that are currently not understood.^73^ We were also limited in our proteome analysis to plasma samples, neglecting cellular contributions to coagulation.^10^ TEG analysis was limited to MA, which is representative of the platelet contribution to clot but also is reflective of a component of fibrinogen.^74^ Cellular protein interactions would not be reflective of platelet-poor protein analysis and could be reflective of why INR captures more proteins associated with massive transfusion than TEG-TIC. These patients were also treated at multiple trauma centers, in which blood samples were obtained in the emergency department. Some of these patients received prehospital blood products. There are multiple unmeasured variables based on centers and patient treatment course that could have impacted the patients measured proteome, limiting the generalizability of these results. Additionally, the samples obtained for these analyses were all obtained from patients who were deemed to be at risk for hemorrhage and TIC by nature of their inclusion in the parent trials. No healthy control or minimally injured patient samples were available to assess baseline proteomic changes in an at risk trauma population. Finally, there are the standard limitations of potential confounding when analyzing nonrandomized data. While the data were from randomized clinical trials, the TIC status was not randomized. These relationships should be interpreted as association and not causation. In addition, these analytes levels have not been validated with ELISA or mass spectrometry, so are at risk of not being accurate measurements.

These data suggest that there are unexplored opportunities to identify patients at risk for massive bleeding. Proteomic analysis using SOMAscan technology suggests that the current laboratory and clinical assessment of TIC may not capture a large number of proteins involved with massive transfusion. This includes known driver of hemostatic derangements involving the fibrinolytic system, DAMPs, and endothelial dysfunction. This hypothesis evaluating study also implicates complement and coagulation factor X as potential mechanisms for prolonged INR, low clot strength, and massive bleeding in trauma.

## Supplementary Material


