# Supplementary material for: Proteomics of Coagulopathy Following Injury Reveals Limitations of Using Laboratory Assessment to Define Trauma-Induced Coagulopathy to Predict Massive Transfusion
Source: Ann Surg Open. 2022 May 25;3(2):e167. doi: 10.1097/AS9.0000000000000167 (PMC9514137; doi:10.1097/AS9.0000000000000167)
Supplement: Supplementary file 6 [file as9-3-e167-s006.pdf]

Supplementary Table 2. Enrichment of functional annotations of non-pathway protein features dysregulated among patients with different indications of TIC.

Functional Annotation	Category	List Hits[1]	List %[1]	Pop Hits[2]	Pop %[2]	Fold Enrichment	FDR[3]
**Massive Transfusion**							
Transmembrane Helix	UniProt Keyword	211	37.5%	387	30.7%	1.22	5.4E-04
Transmembrane	UniProt Keyword	213	37.8%	389	30.8%	1.23	5.8E-04
Receptor	UniProt Keyword	127	22.6%	217	17.2%	1.31	0.0011
Integral Component of Membrane	GO Term: Cellular Compartment	185	32.9%	333	26.5%	1.25	0.0012
Transmembrane Region	UniProt Sequence Feature	194	34.5%	349	27.7%	1.24	0.0014
Topological Domain: Cytoplasmic	UniProt Sequence Feature	186	33.0%	331	26.3%	1.26	0.0019
Topological Domain: Extracellular	UniProt Sequence Feature	174	30.9%	315	25.0%	1.24	0.0087
Membrane	UniProt Keyword	271	48.1%	542	43.0%	1.12	0.065
Glycoprotein	UniProt Keyword	356	63.2%	735	58.3%	1.08	0.073
**INR TIC**							
Signal	UniProt Keyword	163	76.9%	786	62.3%	1.23	2.1E-04
Signal Peptide	UniProt Sequence Feature	162	76.4%	774	61.4%	1.24	4.1E-04
Glycoprotein	UniProt Keyword	148	69.8%	735	58.3%	1.20	0.017
Extracellular Region	GO Term: Cellular Compartment	103	48.6%	473	37.6%	1.30	0.051
Glycosylation Site: N-linked	UniProt Sequence Feature	138	65.1%	677	53.7%	1.21	0.093
**TEG TIC**							
Extracellular Region	GO Term: Cellular Compartment	17	73.9%	473	37.6%	2.06	0.0099
Blood Microparticle	GO Term: Cellular Compartment	7	30.4%	56	4.5%	7.15	0.011
**Clinical TIC**							
None							

[1]**List Hits, List %:** Number and fraction of proteins dysregulated among patients with the given indication of TIC that have the given annotation.
[2]**Pop Hits, Pop %:** Number and fraction of proteins that are targets of SOMAscan aptamers with the given clinical indication of TIC that have the given annotation.
[3]**FDR:** All non-pathway/process terms with a false discovery rate of less than 0.1 are reported here. Pathway terms with FDR < 0.1 are reported in Table 3. Complete results are reported in Appendix Table 5.
